# Running-Induced Fatigue Exacerbates Anteromedial ACL Bundle Stress in Females with Genu Valgum: A Biomechanical Comparison with Healthy Controls

**DOI:** 10.3390/s25154814

**Published:** 2025-08-05

**Authors:** Xiaoyu Jian, Dong Sun, Yufan Xu, Chengyuan Zhu, Xuanzhen Cen, Yang Song, Gusztáv Fekete, Danica Janicijevic, Monèm Jemni, Yaodong Gu

**Affiliations:** 1 Faculty of Sports Science, Ningbo University, Ningbo 315211, China; 2411040010@nbu.edu.cn (X.J.); sundong@nbu.edu.cn (D.S.); 2311040028@nbu.edu.cn (Y.X.); 2211040033@nbu.edu.cn (C.Z.);; 2 Department of Biomedical Engineering, Faculty of Engineering, The Hong Kong Polytechnic University, Hong Kong, China; yangsong@polyu.edu.hk; 3 Department of Material Science and Technology, Audi Hungaria Faculty of Automotive Engineering, Széchenyi István University, 9026 Győr, Hungary; fekete.gusztav@sze.hu; 4 Department of Physical Education and Sport, Faculty of Sport Sciences, University of Granada, 18071 Granada, Spain; jan.danica@nbu.edu.cn; 5 Centre for Mental Health Research in Association with the University of Cambridge, Cambridge CB2 1TN, UK; mjemni@faculty.carrickinstitute.com; 6 The Carrick Institute, Cape Canaveral, FL 32920, USA

**Keywords:** genu valgum, knee model, anterior cruciate ligament, strain, stress

## Abstract

Genu valgum (GV) is a common lower limb deformity that may increase the risk of anterior cruciate ligament (ACL) injury. This study used OpenSim musculoskeletal modeling and kinematic analysis to investigate the mechanical responses of the ACL under fatigue in females with GV. Eight females with GV and eight healthy controls completed a running-induced fatigue protocol. Lower limb kinematic and kinetic data were collected and used to simulate stress and strain in the anteromedial ACL (A–ACL) and posterolateral ACL (P–ACL) bundles, as well as peak joint angles and knee joint stiffness. The results showed a significant interaction effect between group and fatigue condition on A–ACL stress. In the GV group, A–ACL stress was significantly higher than in the healthy group both before and after fatigue (*p* < 0.001) and further increased following fatigue (*p* < 0.001). In the pre-fatigued state, A–ACL strain was significantly higher during the late stance phase in the GV group (*p* = 0.036), while P–ACL strain significantly decreased post-fatigue (*p* = 0.005). Additionally, post-fatigue peak hip extension and knee flexion angles, as well as pre-fatigue knee abduction angles, showed significant differences between groups. Fatigue also led to substantial changes in knee flexion, adduction, abduction, and hip/knee external rotation angles within the GV group. Notably, knee joint stiffness in this group was significantly lower than in controls and decreased further post-fatigue. These findings suggest that the structural characteristics of GV, combined with exercise-induced fatigue, exacerbate A–ACL loading and compromise knee joint stability, indicating a higher risk of ACL injury in fatigued females with GV.

## 1. Introduction

Knee injuries are among the most common sports-related musculoskeletal injuries, accounting for approximately 19.32% of all sports injuries [1]. As a key stabilizing structure of the knee joint [2], the anterior cruciate ligament (ACL) is one of the most frequently injured structures in sports medicine, with more than 70% of cases occurring in non-contact scenarios [3]. ACL injuries not only compromise knee stability and motor function but may also disrupt the normal biomechanics of the joint, leading to changes in joint stiffness. These biomechanical alterations can increase the risk of early-onset osteoarthritis, meniscal damage, and other ligamentous disorders [4,5,6], ultimately leading to long-term joint dysfunction and osteoarthritis [7,8,9]. Non-contact ACL injuries typically occur during rapid movements such as jumping, sudden stops, or changes in direction [10,11] and are often associated with abnormal joint loading, diminished neuromuscular control, and altered movement patterns [12,13].

Genu valgum (GV), a common lower limb structural deformity, has been identified as a significant risk factor for ACL injuries [14]. Individuals with GV demonstrate abnormal lower limb mechanical axis alignment, which leads to increased valgus moment and anterior shear forces at the knee joint during movement, thereby imposing greater mechanical stress on the ACL [15]. These effects are especially pronounced in females. Due to their distinct anatomical structures and differences in neuromuscular control mechanisms, females are more susceptible to exhibiting dynamic knee valgus during physical activity [16]. Consequently, female athletes experience ACL injuries at rates two to eight times higher than their male counterparts [17].

Moreover, exercise-induced fatigue is a key contributing factor to ACL injuries, and its impact may be more pronounced in individuals with GV. Under fatigue, neuromuscular control deteriorates, and muscle strength declines, resulting in reduced knee joint stability [18,19,20]. Curi et al. reported that fatigue may further compromise the stabilizing function of the medial knee muscles in runners with GV, increasing knee valgus angles and thereby elevating shear and torsional loads on the ACL [21]. Other studies have shown that, post-fatigue, individuals tend to land with reduced knee flexion angles and increased vertical ground reaction forces following jumps, which diminishes shock absorption and transfers more impact force directly to the ligaments, thereby increasing the risk of ACL injury [19]. During fatigue, knee adduction moments may increase [22], altering load distribution across the joint and imposing greater tensile and rotational stresses on the ACL [23]. Fatigued female athletes, in particular, are more likely to exhibit movement patterns such as increased knee valgus and reduced knee flexion, both of which are associated with a higher likelihood of ACL injury [17]. In summary, exercise-induced fatigue likely exacerbates ACL injury risk in individuals with GV by impairing neuromuscular control, reducing joint stability, and altering movement patterns.

The combination of genu valgum and exercise-induced fatigue may create a uniquely hazardous condition for the ACL [24]. Genu valgum predisposes the knee to higher valgus loads, and fatigue amplifies this risk [23]. As fatigue progresses, deteriorating neuromuscular control compromises the ability of surrounding muscles to stabilize the joint against these valgus forces [25]. Consequently, the knee must rely more heavily on passive structures, placing significantly greater stress on the ACL.

This loss of dynamic stability is reflected in knee joint stiffness, a measure of the body’s ability to manage joint loads [26]. Fatigue impairs the dynamic adjustment of this stiffness, forcing the ACL to bear loads that muscles would normally handle [27]. This is especially relevant for female athletes, who generally exhibit less capacity to increase knee stiffness compared to males, a factor linked to their higher ACL injury rates [28]. Consequently, in females with genu valgum, a fatigue-induced reduction in knee stiffness may critically impair the joint’s capacity to manage inherent valgus loads, highlighting knee stiffness as a key determinant of their overall injury susceptibility.

Although previous research has extensively explored the impact of sex differences, fatigue, and cutting maneuvers on ACL injury, systematic studies focusing specifically on females with GV under fatigue conditions remain scarce. Filling this research gap is crucial for developing targeted exercise and rehabilitation strategies for individuals with this structural deviation. This study aims to evaluate whether females with GV face a higher risk of ACL injury under fatigue by comparing ACL strain and stress responses pre- and post-exertion between participants with GV and healthy controls. We hypothesize that, under running-induced fatigue, individuals with GV will not only exhibit greater ACL stress and strain but also demonstrate significant alterations in peak hip and knee joint angles. Additionally, knee joint stiffness is expected to decrease markedly and decline further following fatigue, ultimately leading to reduced knee joint stability.

## 2. Materials and Methods

### 2.1. Study Design

In our study, each participant completed all experimental procedures within a single day. The experiment was divided into three phases: pre-fatigue, fatigue protocol, and post-fatigue. Kinematic and kinetic data on the lower limbs were collected during both the pre- and post-fatigue phases. A running-induced fatigue protocol was implemented between the two data collection sessions, and post-fatigue measurements were obtained immediately after the fatigue protocol was completed.

### 2.2. Subjects

**Figure 1:**
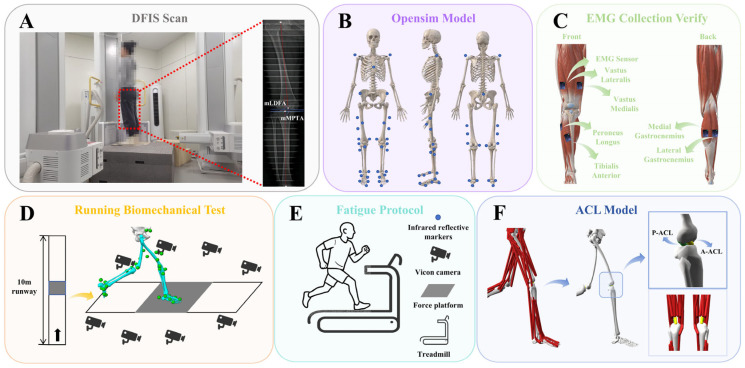
Overview of the musculoskeletal model and the experimental procedure. (**A**) Fluoroscopic knee images were collected and processed using DFIS. (**B**) Locations of reflective markers on the musculoskeletal model. (**C**) Placement of EMG sensors on the lower limbs to validate the model’s accuracy. (**D**) Schematic of the running biomechanics test. (**E**) Diagram of the fatigue protocol. (**F**) Visualization of the ACL physiological model constructed in OpenSim 4.3 (Stanford University, Stanford, CA, USA).

Before the experiment, a power analysis using repeated measures ANOVA was conducted in G·Power (Effect size = 0.4, α = 0.05, power = 0.8, Number of groups = 2, Number of measurements = 2, Corr among rep measurements = 0.5, Nonsphericity correction = 1) [29], which determined that a minimum of 16 participants was required. Accordingly, eight female participants with GV (experimental group) and eight healthy female participants (control group), all aged between 18 and 25 years, were recruited from the student population at Ningbo University. All participants were free of foot deformities and were screened by a physician to exclude any history of lower limb injury or surgery within the past six months. In addition, the GV symptoms in participants of the experimental group were non-traumatic in origin and had been present for at least five years. The participant selection process is outlined in Figure 1. All participants were right-leg dominant and reported regular running activity. Written informed consent was obtained from all participants before data collection, and the study protocol was approved by the Human Ethics Committee of Ningbo University (Approval No. TY2025027).

The GV condition in the experimental group was confirmed using radiographic assessment. A high-speed dual fluoroscopic imaging system (DFIS) was employed to capture static standing images of the knee joint. During image acquisition, participants stood with their knees slightly flexed, and X-ray beams were directed from the anterior superior iliac spine to the inferior edge of the medial malleolus to obtain full-length lower limb joint views (Figure 1A). GV was diagnosed based on two angular measurements: the mechanical lateral distal femoral angle (mLDFA) and the mechanical medial proximal tibial angle (mMPTA) [30]. The mLDFA, defined as the lateral angle between the mechanical axis of the femur and the distal femoral joint surface typically ranges from 85° to 90° [31]. The mMPTA, defined as the medial angle between the tibial mechanical axis and the proximal tibial joint surface (the tibial plateau), also falls within the normal range of 85° to 90° [31]. An mLDFA less than 85° and an mMPTA greater than 90° are indicative of malalignment associated with GV deformity [32]. Participant demographic characteristics are summarized in Table 1.

### 2.3. Experimental Procedures

The experiment was conducted in a 10-m gait laboratory equipped with a nine-camera infrared Vicon motion capture system (Oxford Metrics Ltd., Oxford, UK) and a Kistler force platform (Model 9281B, Winterthur, Switzerland) to collect running motion data. Each participant completed five successful trials, and all wore standardized athletic shoes provided by the laboratory to reduce measurement variability.

Based on previous studies, we collected surface electromyography (sEMG) signals from six muscles, including the tibialis anterior, peroneus longus, vastus lateralis, vastus medialis, and both the medial and lateral heads of the gastrocnemius, using Trigno wireless EMG sensors (Delsys Inc., Boston, MA, USA). Before full-wave rectification, the EMG signals were preprocessed using a 4th-order band-pass filter between 15 and 500 Hz, followed by a 10 Hz low-pass filter to smooth the data. The smoothed EMG signals from six selected muscles were then peak-normalized to standardize muscle activation levels [33], which were subsequently used to validate the accuracy of the musculoskeletal model built in OpenSim (Figure 1C).

Before formal testing, participants’ height and weight were measured using a stadiometer and a calibrated scale. The experimental protocol consisted of three phases: pre-fatigue, fatigue, and post-fatigue. During the pre- and post-fatigue phases, synchronized kinematic and kinetic data were recorded using the EMG sensors, the Vicon motion capture system, and the Kistler force platform (Figure 1D). The sampling frequencies were set at 2000 Hz for EMG, 200 Hz for motion capture, and 2000 Hz for ground reaction force data.

Participants wore form-fitting clothing, and 38 reflective markers were placed on their anatomical landmarks according to the OpenSim 2392 model [34] (Figure 1B). All markers were positioned by the same experienced researcher to ensure consistency. During both pre- and post-fatigue sessions, participants ran at a self-selected comfortable pace [35]. Upon the researcher’s start command, participants ran from the start to the end of the track. Successful data collection required the participant’s right foot to fully contact the force platform. Experimenters assisted in adjusting foot placement to prevent any alteration in gait during the trials.

### 2.4. Running-Induced Fatigue Protocol

After collecting lower limb kinematic and kinetic data under pre-fatigue conditions, participants were instructed to begin the running fatigue protocol (Figure 1E). Fatigue was assessed using the 15-point Borg scale [36], with heart rate continuously monitored via a Polar H10 sensor (Polar Electro Oy, Kempele, Finland). At the start, participants ran at 4 km/h with no incline for a 3-min warm-up. The speed then increased by 1.6 km/h every minute until reaching 12 km/h at 5 min, and this speed was maintained until fatigue onset. Maximum heart rate was estimated using the formula “220 minus age” [37]. Fatigue was defined by meeting all the following criteria simultaneously: (1) subjective feelings of fatigue and difficulty breathing; (2) a rating of perceived exertion of 17; and (3) heart rate reaching 90% of the estimated maximum. To ensure that participants reached a state of physical exhaustion, they were instructed to continue running for an additional 2 min after meeting these criteria. This additional duration was designed to intensify the fatigue response and ensure consistency in the level of running-induced exhaustion across all participants [38].

### 2.5. Data Processing

Marker trajectories were processed using Visual 3D software (version 3.26; C-Motion Inc., Germantown, MD, USA) and smoothed with a low-pass filter set at a 10 Hz cutoff frequency. Joint angles were computed via an inverse kinematics algorithm implemented in Visual 3D. Joint moments were derived from the calculated joint angles and collected ground reaction forces (GRF) using an inverse dynamics algorithm. Knee joint stiffness was calculated as the ratio of knee joint moment to the change in knee joint angle and normalized by the participant’s body weight. Data were normalized to 101 frames per gait cycle, and ground contact was defined when the vertical GRF (VGRF) exceeded 10 N [39].

A musculoskeletal model built in OpenSim (Stanford University, Stanford, CA, USA) was used to simulate and calculate ACL forces [40]. This study focused on changes in ACL stress and strain pre- and post-fatigue in females with GV compared to healthy controls. To accommodate this, additional degrees of freedom (DOF) were added to the frontal and coronal planes of the knee joint when integrating the ACL model into the original OpenSim 2392 model. The ACL’s generalized coordinates, physiological parameters, and DOF functions were based on validated data [41]. For musculoskeletal modeling, individualized skeletal models were first created using Visual3D. Inverse kinematics (IK) was then performed to calculate joint angles during the stance phase of running and to generate motion files (.mot), which were then imported into OpenSim. Following OpenSim guidelines, the Residual Reduction Algorithm (RRA) was applied to reduce modeling errors and inertial inconsistencies caused by marker tracking. Finally, Computed Muscle Control (CMC) and Forward Dynamics (FD) methods were employed to further smooth kinematic data and estimate muscle activations [42]. During data processing, the subtalar and metatarsophalangeal joints were locked [43]. Additionally, ACL stress was normalized to participant body weight, while ACL strain was normalized according to the ratio of ACL length change to its resting length.

Muscle activation data processed using EMG sensors were compared with simulated results from OpenSim musculoskeletal modeling to validate the model’s accuracy and reliability [44]. The agreement between the EMG-measured and simulated muscle activations was evaluated using Pearson correlation analysis [45].

### 2.6. ACL Modeling and Attribute Settings

The smoothed kinematic and kinetic data collected during the running stance phase were processed using Visual 3D and exported as .mot files compatible with OpenSim. These files were subsequently imported into the OpenSim platform to construct a musculoskeletal model focused on the ACL. In this study, the ACL was represented as a nonlinear, elastic soft tissue exhibiting passive mechanical properties [46], as shown in Figure 1F. Anatomically, the ACL originates at the anterior medial intercondylar area of the tibia and inserts on the medial side of the lateral femoral condyle [47]. For biomechanical analysis, the ligament was divided into two main fiber bundles: the anteromedial ACL (A–ACL) and the posterolateral ACL (P–ACL) bundles.

### 2.7. Statistical Analysis

To assess the effects of group (GV vs. control) and fatigue condition (pre-fatigue vs. post-fatigue) on ACL stress and strain during the running stance phase, a two-way repeated-measures ANOVA with statistical parametric mapping (SPM1D) was utilized. This analysis examined the main effects of group and fatigue, as well as the interaction between these factors. In the event of a significant interaction, post-hoc comparisons were performed using a Bonferroni correction to control for Type I error, with the significance level for these specific comparisons adjusted to α < 0.0125. For discrete biomechanical variables, data normality was first assessed with the Shapiro–Wilk test, and the Wilcoxon signed-rank test was subsequently used for non-normally distributed data to compare pre- and post-fatigue conditions. All analyses were conducted using MATLAB (R2024b, The MathWorks Inc., Natick, MA, USA), with an initial significance level of α = 0.05. The SPM1D toolbox (version m.0.4.10) was used for implementation [48] (m.0.4.10, http://www.spm1d.org, accessed on 26 January 2025).

## 3. Results

All statistical results are reported below.

### 3.1. OpenSim Model Verification

**Figure 2:**
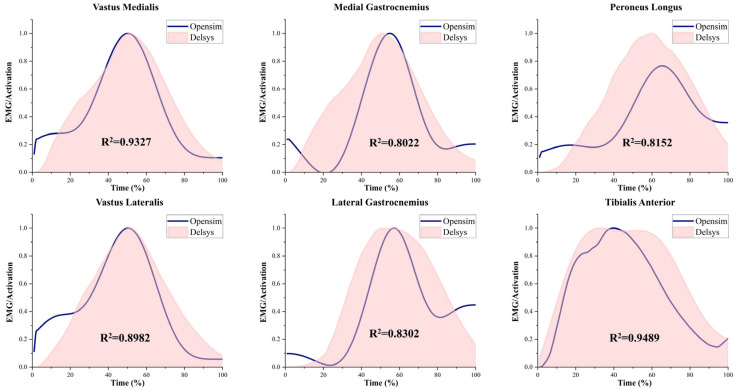
EMG and activation patterns of selected muscles. Simulated activations were generated using the OpenSim-based musculoskeletal model, and experimental EMG data were collected using Delsys sensors. Muscle activations were normalized on a scale from 0 (no activation) to 1 (full activation). The squared correlation coefficient (R^2^) indicates the goodness of fit between simulated and measured data.

Figure 2 presents a comparison between muscle activation data collected via EMG sensors and those derived from musculoskeletal modeling simulations. Both sets of results demonstrated a strong correlation (correlation coefficient > 0.80) [45], indicating a high level of reliability in the simulation outcomes generated from the musculoskeletal model used in this study [44].

### 3.2. ACL Stress Comparison Results

**Figure 3:**
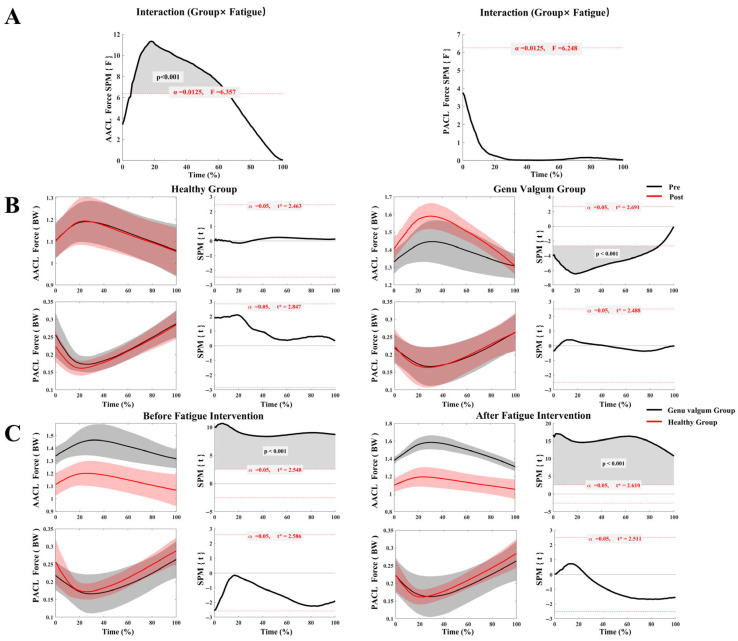
ACL stress in the GV and control groups before and after fatigue. (**A**) Interaction analysis of ACL stress between the GV and control groups across pre- and post-fatigue conditions; (**B**) Comparison of ACL stress before and after fatigue within each group (GV vs. control); (**C**) Comparison of ACL stress between groups (GV vs. control) under different fatigue conditions.

Figure 3A illustrates the interaction effect between group and fatigue condition (pre- and post-fatigue) on ACL stress. The results revealed a significant interaction between group and fatigue condition for A–ACL stress across 9–64% of the stance phase (*p* < 0.001), whereas no interaction effect was found for P–ACL stress. As shown in Figure 3B, the healthy group exhibited no significant differences in A–ACL or P–ACL stress before and after fatigue. In contrast, the GV group showed significantly increased A–ACL stress following fatigue across 1–88% of the stance phase (*p* < 0.001), while no significant difference was observed in P–ACL stress between pre- and post-fatigue conditions. According to Figure 3C, A–ACL stress in the GV group was significantly greater than in the healthy group throughout the entire stance phase, both pre- and post-fatigue (*p* < 0.001). Additionally, P–ACL stress showed only minor differences between groups and did not reach statistical significance.

### 3.3. Comparative Analysis of ACL Strain Results

**Figure 4:**
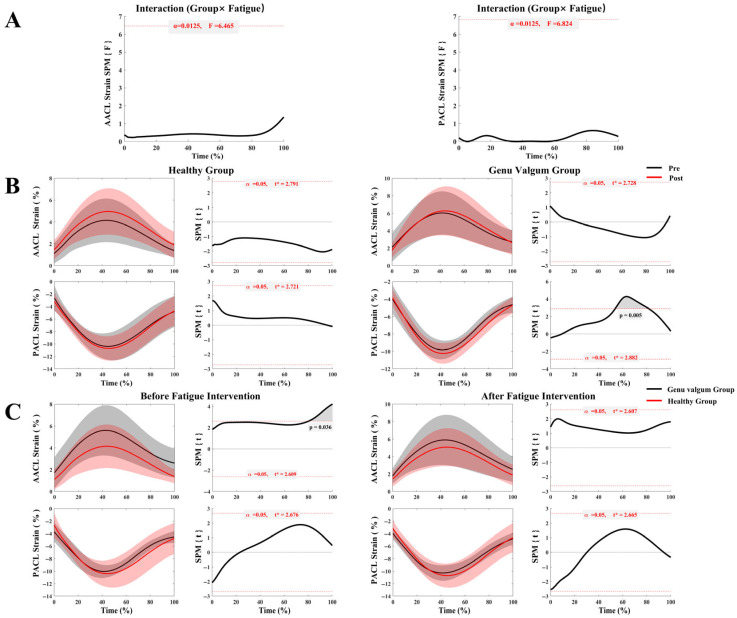
ACL strain in the GV and control groups before and after fatigue. (**A**) Interaction analysis of ACL strain between the GV and control groups across pre- and post-fatigue conditions; (**B**) Comparison of ACL strain before and after fatigue within each group (GV vs. control); (**C**) Comparison of ACL strain between groups (GV vs. control) under different fatigue conditions.

As shown in Figure 4A, there was no significant interaction between group and fatigue condition for the strain on either the A–ACL or P–ACL. Furthermore, Figure 4B indicates that the healthy group exhibited no significant differences in A–ACL or P–ACL strain between pre- and post-fatigue conditions. During the running stance phase, the GV group exhibited no significant change in A–ACL strain with fatigue; however, P–ACL strain significantly decreased post-fatigue compared to before (55–82% of the stance phase, *p* = 0.005). According to Figure 4C, pre-fatigue, A–ACL strain in the GV group was significantly higher than that in the healthy group during 82–100% of the stance phase (*p* = 0.036). Post-fatigue, A–ACL strain remained elevated in the GV group compared to the healthy group throughout the stance phase, but this difference was not statistically significant. Meanwhile, P–ACL strain differences between the two groups pre- and post-fatigue were minimal and not significant.

### 3.4. Comparative Analysis of Lower Limb Joint Angle Peak Values

Table 2 compares the peak joint angles of the hip and knee in the sagittal, coronal, and transverse planes during the running stance phase, pre- and post-fatigue, between the GV and healthy groups. The analysis revealed no significant interaction between group and fatigue condition on peak lower limb joint angles. In the sagittal plane, the GV group exhibited significantly different peak hip extension and peak knee flexion angles post-fatigue compared to the healthy group (*p* < 0.001; *p* = 0.012). Additionally, peak knee flexion angle was significantly greater in the GV group post-fatigue compared to pre-fatigue (*p* = 0.022). In the coronal plane, the GV group showed significant changes in peak knee adduction and abduction angles post-fatigue (*p* = 0.015; *p* = 0.001), with peak knee abduction angle being significantly smaller than that of the healthy group pre-fatigue (*p* = 0.022). In the transverse plane, both peak hip and knee external rotation angles significantly decreased in the GV group following fatigue (*p* = 0.012; *p* < 0.001).

### 3.5. Comparative Analysis of Knee Joint Stiffness Results

Table 3 presents the analysis and statistical comparison of knee joint stiffness during the running support phase between the healthy group and the GV group. The results indicate no interaction effect between group and fatigue condition on knee joint stiffness. However, the healthy group showed significantly higher knee stiffness than the GV group both pre- and post-fatigue (*p* = 0.001; *p* = 0.001). While stiffness decreased in both groups following fatigue, the decline was significantly greater in the GV group (*p* = 0.005).

## 4. Discussion

This study aims to investigate differences in knee joint ACL stress and strain between females with GV and healthy individuals under running-induced fatigue. To analyze these changes, an OpenSim model was employed, representing the ACL as a nonlinear elastic passive tissue. The model’s reliability was validated by collecting EMG data from lower limb muscles and comparing them with CMC results. Consistent with our hypothesis, post-fatigue induced by running, the time pattern of anteromedial ACL stress during the stance phase differed between the GV group and healthy controls, which may increase the risk of ACL injury in the GV group.

The significant interaction effect and the substantially higher A–ACL stress in the GV group, both before and after fatigue, underscore the mechanical disadvantage conferred by a valgus alignment [15]. Structurally, this alignment increases the baseline valgus moment and anterior shear forces on the knee, forcing the A–ACL to sustain higher loads even during non-fatigued movement [24,49,50]. Our results powerfully suggest that running-induced fatigue acts as a critical amplifier of this pre-existing vulnerability. As fatigue degrades neuromuscular control, the muscles’ capacity to dynamically stabilize the joint diminishes [49]. This forces the knee’s passive structures to manage the load [51], with the A–ACL bearing the brunt of these amplified forces [52]. This explains not only the higher baseline stress but also the significant increase in stress observed exclusively in the GV group post-fatigue, painting a clear picture of compounding risk factors.

This study identified a significant reduction in knee joint stiffness in individuals with GV following fatigue. Their knee stiffness decreased notably post-fatigue and was consistently lower than that of healthy controls both pre- and post-fatigue. This suggests that the GV group has compromised knee stability [53], which deteriorates further with fatigue. In contrast, the healthy group exhibited higher knee stiffness, indicating effective cooperation between muscles and the ACL, which helps maintain joint stability and evenly distribute load [54]. Previous research has suggested that the observed post-fatigue increase in A–ACL stress may be linked to weakened joint control and reduced capacity to limit anterior tibial translation after stiffness declines [55]. Additionally, fatigue not only diminishes muscle strength but also delays neuromuscular responses, further weakening knee joint stiffness, a phenomenon that is especially evident in individuals with GV [56,57].

Correspondingly, the divergent strain patterns between the ACL bundles, when viewed alongside kinematic changes, reveal a flawed compensatory mechanism at play, likely adopted in direct response to the loss of dynamic knee stiffness. In the GV group, the significant decrease in P–ACL strain post-fatigue coincided with an increase in their peak knee flexion angle. This shift suggests that under duress, the neuromuscular system prioritizes gross shock absorption via sagittal plane motion over maintaining precise multiplanar joint stability [58,59,60]. However, this adaptation is a double-edged sword. While increased flexion successfully unloads the P–ACL (which is lax in flexion) [49,61], it is a detrimental trade-off that places the A–ACL under greater tension and may alter patellofemoral joint loading patterns [62]. This likely contributes to the higher A–ACL strain observed during the late stance phase pre-fatigue and the elevated stress post-fatigue [24,63]. Therefore, what appears to be a protective adaptation for one ligament bundle inadvertently creates a more hazardous condition for the other, more frequently injured, A–ACL bundle [64]. This fatigue-induced pattern, deeper flexion combined with inherent valgus alignment [62,65], closely mimics established high-risk scenarios for non-contact ACL rupture [66].

Beyond this specific compensatory strategy, the fatigue-induced alterations in other joint angles further highlight a breakdown in multi-planar motor control within the GV group. The significant decrease in peak hip extension suggests a shift toward a more upright and stiffer trunk and lower limb posture, a known risk factor for knee injuries as it limits the body’s ability to dissipate forces through the kinetic chain [67,68]. Concurrently, the significant changes in knee adduction, abduction, and external rotation angles reflect a failing attempt to maintain frontal and transverse plane stability [53]. For a knee already predisposed to valgus collapse [69], this loss of rotational and mediolateral control under fatigue indicates that the neuromuscular system is struggling to counteract the inherent malalignment, leading to erratic joint mechanics and a heightened injury risk [70].

This study reveals a reciprocal interaction between the A–ACL and P–ACL bundles of the ACL. Prior biomechanical research has shown that these two bundles work both complementarily and synergistically to maintain knee stability [50,71,72]. This synergy underlies the tension distribution strategies employed in double-bundle ACL reconstruction [73]. Further investigations indicate that ACL injuries are more frequently observed in the A–ACL bundle [74], likely due to its increased vulnerability to compressive damage from sustained external forces [75]. In contrast, isolated tears of the P–ACL account for 20% to 41% of partial ACL ruptures and typically occur during rotational or directional changes [76]. Isolated A–ACL tears are more prevalent, comprising 59% to 80% of all ACL injuries [76,77]. Such injuries are commonly linked to anterior-directed shear forces acting on the knee, especially during heel strike and the early stance phase of running.

Previous studies have shown that individuals with GV often experience weakness in hip abductor muscles and gluteus maximus, which may increase the load on the A–ACL during movement [17,59]. Therefore, it is recommended that females with GV emphasize strengthening these hip muscles and other core groups that contribute to dynamic knee stability in their regular training [78,79]. Training loads should be carefully managed to avoid sustained high-intensity lower limb activities, such as running, during fatigue. Additionally, neuromuscular training plays a critical role in preventing ACL injuries in this population [80]. Such targeted interventions may improve knee stability and reduce injury risk, offering a valuable alternative exercise strategy.

Although this study provides new insights, several limitations should be noted. The small sample size of only eight females with GV and eight healthy controls restricts the generalizability of the results. Moreover, the analysis was limited to peak joint angles of the hip and knee, excluding ankle joint kinematics. Given the coordinated function of lower limb joints during running, ankle motion may significantly affect knee load distribution and ACL mechanics, warranting inclusion in future research. Furthermore, ACL stress and strain were simulated using OpenSim based on knee kinematics and ground reaction forces to estimate ligament length changes. Despite constructing a comprehensive musculoskeletal model validated by electromyography, the simulation simplifies the complex double-bundle structure of the ACL. This simplification may overlook interactions between the A–ACL and P–ACL and individual anatomical differences. Although EMG data were collected, the study did not explore EMG-based fatigue metrics such as median frequency shifts or zero-crossing rate, which could offer more objective indicators of neuromuscular fatigue [81]. Future studies may consider incorporating such methods for more comprehensive fatigue assessment. Lastly, finite element analysis has been extensively used in sports biomechanics to simulate the impact of equipment on human tissues [82,83,84,85]. Future studies should integrate finite element analysis and high-resolution medical imaging to acquire individualized ACL data and develop high-fidelity ligament models. Such advancements would enable more accurate simulations of ACL stress and strain across various movements, providing deeper insight into injury mechanisms.

## 5. Conclusions

This study employed OpenSim modeling to analyze changes in ACL stress and strain in females with GV under fatigued conditions. The results indicated a significant interaction between group and fatigue condition in A–ACL stress. Compared to healthy individuals, the GV group experienced significantly greater A–ACL stress both before and after fatigue, along with a marked reduction in knee joint stiffness. Moreover, A–ACL stress increased significantly after fatigue within the GV group. Under pre-fatigued conditions, the GV group showed significantly greater A–ACL strain at the end of the running stance phase compared to controls, while P–ACL strain decreased significantly following fatigue. Significant differences in peak knee flexion angle post-fatigue were also observed between the GV and healthy groups. In addition, peak knee flexion, adduction, abduction, and external rotation angles in the GV group changed significantly post-fatigue relative to pre-fatigue values. These findings highlight the combined influence of valgus knee structural abnormalities and fatigue on the mechanical loading of the ACL. Future studies should utilize larger sample sizes along with high-resolution imaging and finite element modeling to enhance the precision of ACL stress and strain estimates. Moreover, investigating targeted muscle training interventions could inform the development of scientifically grounded injury prevention programs for individuals with GV.

## Figures and Tables

**Table 1:** Overview of Demographic Characteristics.

Variables	GV Group		Healthy Group	
	Mean	SD	Mean	SD
Age (year)	23.0	1.4	23.0	3.2
Weight (kg)	59.1	11.1	50.3	3.6
Height (cm)	163.7	8.7	161.6	3.5
BMI	21.9	1.9	19.3	1.6
mLDFA	82.3	0.4	87.2	0.3
mMPTA	91.6	0.3	87.8	0.7
Length of the A–ACL (mm)	23.1	2.2	23.3	1.4
Length of the P–ACL (mm)	30.1	1.6	30.0	0.9

Notes: BMI: body mass index; SD: Standard deviation.

**Table 2:** Comparison of Peak Joint Angles at the Hip and Knee Between the GV and Control Groups.

	Variables	GV Group		Healthy Group		*p*
	Peak Angle	Pre-Fatigue	Post-Fatigue	Pre-Fatigue	Post-Fatigue	
Sagittal Plane	Hip Flexion	44.37 ± 4.44	45.65 ± 5.96	45.98 ± 8.75	45.68 ± 5.17	0.06
	Hip Extension	−1.86 ± 6.33	−1.42 ± 5.90	3.63 ± 3.95	3.80 ± 2.69 #	0.08
	Knee Flexion	37.45 ± 4.93	41.75 ± 6.98 *	35.16 ± 6.17	37.17 ± 7.49 #	0.43
	Knee Extension	−17.01 ± 4.24	−18.12 ± 5.77	−9.38 ± 5.49	−8.06 ± 2.60	0.22
Frontal Plane	Hip Adduction	7.94 ± 3.36	8.37 ± 2.44	10.09 ± 6.47	10.78 ± 6.47	0.88
	Hip Abduction	3.62 ± 4.74	4.17 ± 3.85	5.39 ± 1.75	4.53 ± 2.44	0.49
	Knee Adduction	4.71 ± 3.85	3.12 ± 4.47 *	2.90 ± 3.19	2.33 ± 3.23	0.77
	Knee Abduction	4.54 ± 3.39	5.77 ± 5.28 *	6.10 ± 3.23 #	6.82 ± 3.68	0.37
Transverse Plane	Hip Internal Rotation	15.56 ± 5.73	16.71 ± 4.84	18.26 ± 5.77	19.08 ± 2.55	0.54
	Hip External Rotation	5.18 ± 7.65	3.30 ± 9.85 *	5.19 ± 9.07	5.16 ± 6.47	0.73
	Knee Internal Rotation	17.20 ± 6.51	20.95 ± 2.45	20.37 ± 5.10	21.21 ± 4.23	0.78
	Knee External Rotation	19.89 ± 4.24	16.86 ± 7.23 *	17.32 ± 5.71	16.53 ± 4.30	0.69

Notes: Outcome variables are shown as the means ± standard deviations. Significant differences within groups: * *p*  <  0.05; Significant differences between groups: # *p* <  0.05.

**Table 3:** Analysis of knee joint stiffness (N·m/kg·°).

Variables	Healthy Group	GV Group	*p* _Group_	*p*
Pre-fatigue	0.043 ± 0.020	0.034 ± 0.013	0.001	0.073
Post-fatigue	0.041 ± 0.018	0.029 ± 0.022	0.001	
*p* _Fatigue_	0.063	0.007		

Notes: Outcome variables are shown as the means ± standard deviations. *p* Fatigue: Statistical significance difference in knee joint stiffness between pre- and post-fatigue; *p* Group: Statistical significance difference in knee joint stiffness between the healthy group and GV group.

## Data Availability

The data presented in this study are available on request from the corresponding author. The data are not publicly available due to ethical considerations.

## References

[1] Prieto-González P., Martínez-Castillo J.L., Fernández-Galván L.M., Casado A., Soporki S., Sánchez-Infante J. (2021). Epidemiology of Sports-Related Injuries and Associated Risk Factors in Adolescent Athletes: An Injury Surveillance. Int. J. Environ. Res. Public Health.

[2] Wang B., Zhong J.-L., Xu X.-H., Shang J., Lin N., Lu H.-D. (2020). Incidence and risk factors of joint stiffness after anterior cruciate ligament reconstruction. J. Orthop. Surg. Res..

[3] Griffin L.Y., Agel J., Albohm M.J., Arendt E.A., Dick R.W., Garrett W.E., Garrick J.G., Hewett T.E., Huston L., Ireland M.L. (2000). Noncontact anterior cruciate ligament injuries: Risk factors and prevention strategies. J. Am. Acad. Orthop. Surg..

[4] Simon D., Mascarenhas R., Saltzman B.M., Rollins M., Bach B.R., MacDonald P. (2015). The Relationship between Anterior Cruciate Ligament Injury and Osteoarthritis of the Knee. Adv. Orthop..

[5] Tayton E., Verma R., Higgins B., Gosal H. (2009). A correlation of time with meniscal tears in anterior cruciate ligament deficiency: Stratifying the risk of surgical delay. Knee Surg. Sports Traumatol. Arthrosc..

[6] Gelber A.C., Hochberg M.C., Mead L.A., Wang N.Y., Wigley F.M., Klag M.J. (2000). Joint injury in young adults and risk for subsequent knee and hip osteoarthritis. Ann. Intern. Med..

[7] Arendt E., Dick R. (1995). Knee injury patterns among men and women in collegiate basketball and soccer: NCAA data and review of literature. Am. J. Sports Med..

[8] Wetters N., Weber A.E., Wuerz T.H., Schub D.L., Mandelbaum B.R. (2016). Mechanism of injury and risk factors for anterior cruciate ligament injury. Oper. Tech. Sports Med..

[9] Pfeifer C.E., Beattie P.F., Sacko R.S., Hand A. (2018). Risk factors associated with non-contact anterior cruciate ligament injury: A systematic review. Int. J. Sports Phys. Ther..

[10] Sell T.C., Ferris C.M., Abt J.P., Tsai Y.-S., Myers J.B., Fu F.H., Lephart S.M. (2006). The effect of direction and reaction on the neuromuscular and biomechanical characteristics of the knee during tasks that simulate the noncontact anterior cruciate ligament injury mechanism. Am. J. Sports Med..

[11] Li F., Sun D., Song Y., Fang Y., Cen X., Zhang Q., Gu Y. (2025). Comparison of Landing Biomechanics in Male Amateur Basketball Players with and without Patellar Tendinopathy during Simulated Games. J. Hum. Kinet..

[12] Li F., Song Y., Cen X., Sun D., Lu Z., Bíró I., Gu Y. (2023). Comparative efficacy of vibration foam rolling and cold water immersion in amateur basketball players after a simulated load of basketball game. Healthcare.

[13] Wang D., Sun D., Zhou Z., Li F., Cen X., Song Y., Gu Y. (2024). Comparison of stop-jump muscle synergies in amateur basketball players with and without asymptomatic patellar tendon abnormalities during simulated games. Acta Bioeng. Biomech..

[14] Ashraf S., Viveiros R., França C., Ornelas R.T., Rodrigues A. (2024). Association between Body Composition, Physical Activity Profile, and Occurrence of Knee and Foot Postural Alterations among Young Healthy Adults. Future.

[15] Larwa J., Stoy C., Chafetz R.S., Boniello M., Franklin C. (2021). Stiff Landings, Core Stability, and Dynamic Knee Valgus: A Systematic Review on Documented Anterior Cruciate Ligament Ruptures in Male and Female Athletes. Int. J. Environ. Res. Public Health.

[16] Khayambashi K., Ghoddosi N., Straub R.K., Powers C.M. (2016). Hip Muscle Strength Predicts Noncontact Anterior Cruciate Ligament Injury in Male and Female Athletes: A Prospective Study. Am. J. Sports Med..

[17] Mancino F., Kayani B., Gabr A., Fontalis A., Plastow R., Haddad F.S. (2024). Anterior cruciate ligament injuries in female athletes: Risk factors and strategies for prevention. Bone Jt. Open.

[18] Ortiz A., Olson S.L., Etnyre B., Trudelle-Jackson E.E., Bartlett W., Venegas-Rios H.L. (2010). Fatigue effects on knee joint stability during two jump tasks in women. J. Strength Cond. Res..

[19] Wong T.L., Huang C.F., Chen P.C. (2020). Effects of Lower Extremity Muscle Fatigue on Knee Loading During a Forward Drop Jump to a Vertical Jump in Female Athletes. J. Hum. Kinet..

[20] Jiang X., Sárosi J., Bíró I. (2024). Characteristics of lower limb running-related injuries in trail runners: A systematic review. Phys. Act. Health.

[21] Curi G., Costa F.D.D., Medeiros V.S., Barbosa V.D., Santos T.R.T., Dionisio V.C. (2024). The effects of core muscle fatigue on lower limbs and trunk during single-leg drop landing: A comparison between recreational runners with and without dynamic knee valgus. Knee.

[22] Benjaminse A., Webster K.E., Kimp A., Meijer M., Gokeler A. (2019). Revised Approach to the Role of Fatigue in Anterior Cruciate Ligament Injury Prevention: A Systematic Review with Meta-Analyses. Sports Med..

[23] Chappell J.D., Herman D.C., Knight B.S., Kirkendall D.T., Garrett W.E., Yu B. (2005). Effect of fatigue on knee kinetics and kinematics in stop-jump tasks. Am. J. Sports Med..

[24] Hewett T.E., Myer G.D., Ford K.R., Heidt R.S., Colosimo A.J., McLean S.G., Van den Bogert A.J., Paterno M.V., Succop P. (2005). Biomechanical measures of neuromuscular control and valgus loading of the knee predict anterior cruciate ligament injury risk in female athletes: A prospective study. Am. J. Sports Med..

[25] Quatman C.E., Hewett T.E. (2009). The anterior cruciate ligament injury controversy: Is “valgus collapse” a sex-specific mechanism?. Br. J. Sports Med..

[26] Wang D., de Vito G., Ditroilo M., Delahunt E. (2017). Neuromuscular training effects on the stiffness properties of the knee joint and landing biomechanics of young female recreational athletes. Br. J. Sports Med..

[27] Bencke J., Aagaard P., Zebis M.K. (2018). Muscle Activation During ACL Injury Risk Movements in Young Female Athletes: A Narrative Review. Front. Physiol..

[28] Ireland M.L. (2002). The female ACL: Why is it more prone to injury?. Orthop. Clin..

[29] Borgia B., Dufek J.S., Silvernail J.F., Radzak K.N. (2022). The effect of fatigue on running mechanics in older and younger runners. Gait Posture.

[30] Marques Luís N., Varatojo R. (2021). Radiological assessment of lower limb alignment. EFORT Open Rev..

[31] Paley D. (2014). Principles of Deformity Correction.

[32] Farr S., Kranzl A., Pablik E., Kaipel M., Ganger R. (2014). Functional and radiographic consideration of lower limb malalignment in children and adolescents with idiopathic genu valgum. J. Orthop. Res..

[33] Besomi M., Hodges P.W., Clancy E.A., Van Dieën J., Hug F., Lowery M., Merletti R., Søgaard K., Wrigley T., Besier T. (2020). Consensus for experimental design in electromyography (CEDE) project: Amplitude normalization matrix. J. Electromyogr. Kinesiol..

[34] Gao S., Song Y., Sun D., Cen X., Wang M., Lu Z., Gu Y. (2025). Impact of Becker muscular dystrophy on gait patterns: Insights from biomechanical analysis. Gait Posture.

[35] Chang H., Cen X. (2024). Can running technique modification benefit patellofemoral pain improvement in runners? A systematic review and meta-analysis. Int. J. Biomed. Eng. Technol..

[36] Williams N. (2017). The Borg Rating of Perceived Exertion (RPE) scale. Occup. Med..

[37] Liguori G., American College of Sports Medicine (ACSM) (2020). ACSM’s Guidelines for Exercise Testing and Prescription.

[38] Yu P., Gong Z., Meng Y., Baker J.S., István B., Gu Y. (2020). The acute influence of running-induced fatigue on the performance and biomechanics of a countermovement jump. Appl. Sci..

[39] Cen X., Yu P., Song Y., Sun D., Liang M., Bíró I., Gu Y. (2024). Influence of medial longitudinal arch flexibility on lower limb joint coupling coordination and gait impulse. Gait Posture.

[40] Li F., Sun D., Song Y., Zhou Z., Wang D., Cen X., Gu Y. (2025). Dynamic simulation of knee joint mechanics: Individualized multi-moment finite element modelling of patellar tendon stress during landing. J. Biomech..

[41] Kar J., Quesada P.M. (2012). A numerical simulation approach to studying anterior cruciate ligament strains and internal forces among young recreational women performing valgus inducing stop-jump activities. Ann. Biomed. Eng..

[42] Thelen D.G., Anderson F.C., Delp S.L. (2003). Generating dynamic simulations of movement using computed muscle control. J. Biomech..

[43] Rajagopal A., Dembia C.L., DeMers M.S., Delp D.D., Hicks J.L., Delp S.L. (2016). Full-body musculoskeletal model for muscle-driven simulation of human gait. IEEE Trans. Biomed. Eng..

[44] Song Y., Cen X., Wang M., Gao Z., Tan Q., Sun D., Gu Y., Wang Y., Zhang M. (2025). A Systematic Review of Finite Element Analysis in Running Footwear Biomechanics: Insights for Running-Related Musculoskeletal Injuries. J. Sports Sci. Med..

[45] Schober P., Boer C., Schwarte L.A. (2018). Correlation coefficients: Appropriate use and interpretation. Anesth. Analg..

[46] Cheng E.J., Brown I.E., Loeb G.E. (2000). Virtual muscle: A computational approach to understanding the effects of muscle properties on motor control. J. Neurosci. Methods.

[47] Sikidar A., Marieswaran M., Kalyanasundaram D. (2021). Estimation of forces on anterior cruciate ligament in dynamic activities. Biomech. Model. Mechanobiol..

[48] Pataky T.C., Vanrenterghem J., Robinson M. Statistical parametric mapping (SPM): Theory, software, and future directions. Proceedings of the International Society of Biomechanics.

[49] Amis A.A., Dawkins G. (1991). Functional anatomy of the anterior cruciate ligament. Fibre bundle actions related to ligament replacements and injuries. J. Bone Jt. Surg. Br. Vol..

[50] Gabriel M.T., Wong E.K., Woo S.L.Y., Yagi M., Debski R.E. (2004). Distribution of in situ forces in the anterior cruciate ligament in response to rotatory loads. J. Orthop. Res..

[51] Yu B., Lin C.F., Garrett W.E. (2006). Lower extremity biomechanics during the landing of a stop-jump task. Clin. Biomech..

[52] Markolf K.L., Burchfield D.M., Shapiro M.M., Shepard M.F., Finerman G.A., Slauterbeck J.L. (1995). Combined knee loading states that generate high anterior cruciate ligament forces. J. Orthop. Res..

[53] Schmitz R.J., Ficklin T.K., Shimokochi Y., Nguyen A.D., Beynnon B.D., Perrin D.H., Shultz S.J. (2008). Varus/valgus and internal/external torsional knee joint stiffness differs between sexes. Am. J. Sports Med..

[54] Zlotnicki J.P., Naendrup J.H., Ferrer G.A., Debski R.E. (2016). Basic biomechanic principles of knee instability. Curr. Rev. Musculoskelet. Med..

[55] Wu D., Zhao X., Wu B., Zhou L., Luo Y., Huang X., Xu W., Wang S. (2023). Subregional analysis of joint stiffness facilitates insight into ligamentous laxity after ACL injury. Front. Bioeng. Biotechnol..

[56] Wang D., de Vito G., Ditroilo M., Delahunt E. (2017). Different Effect of Local and General Fatigue on Knee Joint Stiffness. Med. Sci. Sports Exerc..

[57] Shao E., Lu Z., Cen X., Zheng Z., Sun D., Gu Y. (2022). The Effect of Fatigue on Lower Limb Joint Stiffness at Different Walking Speeds. Diagnostics.

[58] Saber B., Bridger D., Agrawal D.K. (2024). A Critical Analysis of the Factors Contributing to Anterior Cruciate Ligament Injuries in Female Athletes. J. Orthop. Sports Med..

[59] Rinaldi V.G., Prill R., Jahnke S., Zaffagnini S., Becker R. (2022). The influence of gluteal muscle strength deficits on dynamic knee valgus: A scoping review. J. Exp. Orthop..

[60] Khou S.B., Saki F., Tahayori B. (2024). Muscle activation in the lower limb muscles in individuals with dynamic knee valgus during single-leg and overhead squats: A meta-analysis study. BMC Musculoskelet. Disord..

[61] Takai S., Woo S.L., Livesay G.A., Adams D.J., Fu F.H. (1993). Determination of the in situ loads on the human anterior cruciate ligament. J. Orthop. Res..

[62] Hashemi J., Breighner R., Chandrashekar N., Hardy D.M., Chaudhari A.M., Shultz S.J., Slauterbeck J.R., Beynnon B.D. (2011). Hip extension, knee flexion paradox: A new mechanism for non-contact ACL injury. J. Biomech..

[63] Withrow T.J., Huston L.J., Wojtys E.M., Ashton-Miller J.A. (2006). The effect of an impulsive knee valgus moment on in vitro relative ACL strain during a simulated jump landing. Clin. Biomech..

[64] Shao E., Mei Q., Ye T., Kovács B., Baker J.S., Liu W., Gu Y. (2023). The Effects of 5 km Interval Running on the Anterior Cruciate Ligament Strain and Biomechanical Characteristic of the Knee Joint: Simulation and Principal Component Analysis. Appl. Sci..

[65] Yoon S.W., Lee J.W., Cho W.S., Kim A.N., Lee K.H. (2013). Analysis of balance ability dependent on the angle of the knee joint in females in their 20s. J. Phys. Ther. Sci..

[66] Shimokochi Y., Shultz S.J. (2008). Mechanisms of noncontact anterior cruciate ligament injury. J. Athl. Train..

[67] Barrios J.A., Heitkamp C.A., Smith B.P., Sturgeon M.M., Suckow D.W., Sutton C.R. (2016). Three-dimensional hip and knee kinematics during walking, running, and single-limb drop landing in females with and without genu valgum. Clin. Biomech..

[68] Blackburn J.T., Padua D.A. (2008). Influence of trunk flexion on hip and knee joint kinematics during a controlled drop landing. Clin. Biomech..

[69] Baird D.C., Dickison C.G., Spires H.I. (2025). Lower Extremity Abnormalities in Children. Am. Fam. Physician.

[70] Hinckel B.B., Demange M.K., Gobbi R.G., Pécora J.R., Camanho G.L. (2016). The Effect of Mechanical Varus on Anterior Cruciate Ligament and Lateral Collateral Ligament Stress: Finite Element Analyses. Orthopedics.

[71] Girgis F.G., Marshall J.L., JEM A.A.M. (1975). The cruciate ligaments of the knee joint: Anatomical, functional and experimental analysis. Clin. Orthop. Relat. Res..

[72] Zantop T., Herbort M., Raschke M.J., Fu F.H., Petersen W. (2007). The role of the anteromedial and posterolateral bundles of the anterior cruciate ligament in anterior tibial translation and internal rotation. Am. J. Sports Med..

[73] Colombet P., Robinson J., Jambou S., Allard M., Bousquet V., de Lavigne C. (2006). Two-bundle, four-tunnel anterior cruciate ligament reconstruction. Knee Surg. Sports Traumatol. Arthrosc..

[74] Grunenberg O., Gerwing M., Oeckenpöhler S., Peez C., Briese T., Glasbrenner J., Hägerich L.M., Raschke M.J., Kittl C., Herbst E. (2024). The anteromedial retinaculum in ACL-injured knees: An overlooked injury?. Knee Surg. Sports Traumatol. Arthrosc..

[75] Yeo I.-S., Hong J.-E., Yang H.-M. (2025). Histomorphometric analysis of anterior cruciate ligament bundles and anatomical insights into injury mechanisms. Sci. Rep..

[76] Yadav S., Singh S. (2020). Analysis of partial bundle anterior cruciate ligament tears- diagnosis and management with ACL augmentation. J. Clin. Orthop. Trauma..

[77] Beaulieu M.L., Ashton-Miller J.A., Wojtys E.M. (2023). Loading mechanisms of the anterior cruciate ligament. Sports Biomech..

[78] Bien D.P. (2011). Rationale and implementation of anterior cruciate ligament injury prevention warm-up programs in female athletes. J. Strength Cond. Res..

[79] Maniar N., Cole M.H., Bryant A.L., Opar D.A. (2022). Muscle Force Contributions to Anterior Cruciate Ligament Loading. Sports Med..

[80] Moshashaei M.S., Gandomi F., Amiri E., Maffulli N. (2024). Anodal tDCS improves the effect of neuromuscular training on the feedforward activity of lower extremity muscles in female taekwondo athletes with dynamic knee valgus. Sci. Rep..

[81] Kim H., Lee J., Kim J. (2018). Electromyography-signal-based muscle fatigue assessment for knee rehabilitation monitoring systems. Biomed. Eng. Lett..

[82] Sun D., Song Y., Cen X., Wang M., Baker J.S., Gu Y. (2022). Workflow assessing the effect of Achilles tendon rupture on gait function and metatarsal stress: Combined musculoskeletal modeling and finite element analysis. Proc. Inst. Mech. Eng. Part H J. Eng. Med..

[83] Song Y., Cen X., Wang M., Bálint K., Tan Q., Sun D., Gao S., Li F., Gu Y., Wang Y. (2025). The influence of simulated worn shoe and foot inversion on heel internal biomechanics during running impact: A subject-specific finite element analysis. J. Biomech..

[84] Song Y., Cen X., Sun D., Bálint K., Wang Y., Chen H., Gao S., Biro I., Zhang M., Gu Y. (2024). Curved carbon-plated shoe may further reduce forefoot loads compared to flat plate during running. Sci. Rep..

[85] Cen X., Song Y., Sun D., Bíró I., Gu Y. (2023). Applications of finite element modeling in biomechanical analysis of foot arch deformation: A scoping review. J. Biomech. Eng..

